# Profiles of exercise adherence in late pregnancy: a latent profile analysis among Chinese women

**DOI:** 10.3389/fpubh.2026.1838413

**Published:** 2026-05-20

**Authors:** He Ma, Huina Chen, Lifen Ouyang, Shuru Xu, Zhaomei Xie, Keqing Li, Dongmei Duan, Chenhui Zhou

**Affiliations:** 1 School of Nursing, Guangdong Medical University, Dongguan, Guangdong, China; 2 Department of Nursing, Shunde Women and Children’s Hospital of Guangdong Medical University, Foshan, Guangdong, China; 3 Department of Nursing, Dongguan Maternal and Child Health Care Hospital, Dongguan, Guangdong, China

**Keywords:** exercise adherence, latent profile analysis, pregnant women, self-efficacy, social support

## Abstract

**Background:**

The advantages of physical activity and exercise interventions for pregnant women largely rely on sustained adherence. This study aimed to identify latent categories of exercise adherence among women in the third trimester of pregnancy and to examine the factors associated with these distinct profiles.

**Methods:**

Convenience sampling was applied to recruit participants from three maternal and child health hospitals in China between November 2024 and June 2025. Latent profile analysis (LPA) was employed to determine potential classes of exercise adherence among pregnant women in the third trimester, and multinomial logistic regression was conducted to examine factors related to these profiles.

**Results:**

A total of 531 participants were enrolled in this study. Of them, only 23.4% achieved the recommended exercise level and were categorized as having high (*n* = 59), moderate (*n* = 239), and low adherence (*n* = 233) to exercise. Compared with the high-adherence group, the associated factors for the low-adherence group included weekly exercise habits, pelvic floor muscle training (PFMT), self-efficacy, social support, enjoyment of exercise, competing commitments, limited time, and fatigue. In contrast, those for the mid-adherence group were education level, weekly exercise habits, self-efficacy, social support, lack of time, and fatigue.

**Conclusion:**

Exercise adherence among pregnant women can be grouped into three distinct categories. Health professionals should design targeted interventions according to the socio-psychological characteristics of women in the third trimester to enhance their adherence to exercise.

## 1 Introduction

Pregnancy represents a physiologically and psychologically distinctive period that is vital for the health of pregnant women and their offspring. Regular engagement in physical activity and exercise during pregnancy is widely acknowledged to improve maternal well-being and support optimal development of offspring (1). Although the World Health Organization (WHO) recommends that women with healthy pregnancies perform at least 150 min of moderate-intensity aerobic exercise per week (2), adherence remains a critical determinant of the effectiveness of exercise during pregnancy. Evidence indicates that adherence to these recommended guidelines is often insufficient, thereby restricting the potential health benefits that may be achieved (3).

Previous investigations have suggested that physical inactivity during pregnancy increases the likelihood of excessive gestational weight gain, gestational diabetes mellitus, pre-eclampsia, gestational hypertension, macrosomia, instrumental delivery, urinary incontinence, and depressive disorders (4, 5). Sarno et al. (6) recruited women with a singleton and uncomplicated pregnancy during the third trimester, and 37% of participants reported engaging in sports or exercise activities. A systematic review involving 11,323 Chinese women during pregnancy found that only 21.0% achieved the recommended level of exercise (7). A cross-sectional study including 1,636 Chinese pregnant women showed that the prevalence of physical inactivity was 47.5%, and walking was identified as the most frequently performed type of physical activity (8).

The benefits of physical activity and exercise for both mother and child depend largely on sustained adherence over time. Walasik et al. (9) investigated patterns of physical activity among 9,000 pregnant women in Poland. During the first and second trimesters, about 90% of participants reported exercising, whereas in the later stage of pregnancy, nearly 13% discontinued physical activity. Kókai et al. (10) evaluated the effectiveness of two 8-week app-based moderate-to-vigorous physical activity interventions designed for pregnant women. This study commenced in October 2021 with 663 participants. By week 21, 254 women had withdrawn from the intervention program. Shang et al. (11) reported that fewer than half of the women performed 150 min of exercise per week before pregnancy, and only 40.5% of participants maintained regular exercise according to recommended guidelines during pregnancy.

Adherence, as defined by the WHO, refers to the extent to which an individual’s behavior corresponds with agreed recommendations provided by health care professionals (12). Exercise adherence during pregnancy is a multifaceted and dynamic phenomenon that is influenced by demographic, psychological, and environmental determinants (11). Previous studies mainly adopted a variable-centered approach to classify pregnant women according to whether their physical activity and exercise met the recommended standards (6). Participants were often divided into “exercisers” and “non-exercisers” depending on whether they engaged in recommended levels of physical activity in order to assess adherence to exercise (3, 6, 10, 13). However, such analytical approaches may be insufficient for guiding individualized interventions. Furthermore, knowledge regarding the reasons underlying adherence to exercise during pregnancy remains relatively limited.

Exercise adherence, together with reasons for both adherence and non-adherence, was measured using the Exercise Adherence Rating Scale (EARS) (14), a reliable and validated self-reported instrument consisting of 6 items assessing adherence to exercise and 10 items evaluating reasons related to exercise adherence (15). The EARS has been translated and applied in several countries, including Denmark (16), Brazil (17), and Sweden (18). In the present study, adherence to physical activity among low-risk pregnant women was assessed by adapting a Chinese version of the EARS.

A comprehensive understanding of person-centered factors that influence physical activity during pregnancy is essential for developing effective and individualized exercise programs. Latent profile analysis (LPA), a statistical method particularly suitable for identifying underlying individual characteristics from a person-centered perspective, was further employed. The findings could contribute to the development of tailored exercise interventions for pregnant women.

To the best of our knowledge, no previous research has applied LPA to investigate patterns of exercise adherence among pregnant women. Therefore, the present study used LPA to identify adherence profiles among pregnant women from three cities in Guangdong—Shenzhen, Dongguan, and Shunde (Foshan)—and to explore factors associated with these profiles.

## 2 Materials and methods

### 2.1 Study design and participants

An observational study was carried out between November 2024 and June 2025 at three maternal and child health hospitals located in Shenzhen, Dongguan, and Shunde in Guangdong Province, China. Convenience sampling was adopted to recruit pregnant women who were in the third trimester of pregnancy. The inclusion criteria were as follows: women aged ≥ 18 years and those willing to participate voluntarily in the study. The exclusion criteria were defined as: age <18 years; presence of significant chronic conditions that might influence exercise during pregnancy (e.g., pre-eclampsia, cervical insufficiency, unexplained persistent vaginal bleeding); diagnosed mental disorders; or refusal to participate.

Data were collected in person by six trained researchers. After confirming eligibility, the researchers explained the objectives, potential risks, and expected benefits of the study to the participants. Women who satisfied the inclusion criteria completed the questionnaire immediately, which helped reduce the probability of incomplete or invalid responses.

### 2.2 Sampling method and sample size

According to the literature (16), latent profile analysis generally requires a minimum sample size ranging from 250 to 500 participants. Therefore, this study adopted the LPA approach and followed established recommendations for determining an adequate sample size. A total of 531 participants were finally recruited from 558 eligible pregnant women using convenience sampling.

### 2.3 Measures

A structured questionnaire was developed based on a review of relevant literature and the objectives of the study. This instrument collected information on several demographic characteristics, including age, pre-pregnancy body mass index (BMI), place of residence, education level, personal monthly income, and other related variables. Among these indicators, pre-pregnancy BMI was calculated by dividing body weight by the square of height. According to the Chinese classification criteria, women with a BMI < 18.5 kg/m^2^ are categorized as underweight, those with a BMI of 18.5–23.9 kg/m^2^ as normal weight, those with a BMI of 24–27.9 kg/m^2^ as overweight, and those with a BMI ≥ 28 kg/m^2^ as obese (17). Obstetric characteristics included information on the history of gravidity and parity.

### 2.4 Pilot study

To ensure study quality, a pilot test was conducted from September to October 2024 at three hospitals in Shenzhen, Dongguan, and Shunde, Guangdong Province, China. Each hospital had one data collector. All included questionnaires were completed in more than 300 s. A total of 197 questionnaires were collected, of which 182 were valid, and the questionnaire response rate of pregnant women was 92.38%. The questionnaire showed good reliability and validity.

### 2.5 Exercise adherence

Exercise adherence was evaluated using the Exercise Adherence Rating Scale (EARS) developed by Newman in 2017 (14). This instrument comprises three sections: the EARS-A scale includes 6 qualitative questions that provide information about adherence behavior in individuals; the EARS-B scale contains 6 items assessing adherence to prescribed home exercise, in which items 1, 3, and 5 are reverse scored; and the EARS-C scale consists of 10 questions that explore reasons for adherence or non-adherence, with items 3, 7, 8, and 10 reverse scored (14, 15). The scale has been translated and validated in several countries and has demonstrated good reliability and validity (Cronbach’s *α* = 0.77 to 0.94) (18–20). The Chinese version of EARS was revised by Wu et al. (15). The total score of the EARS-B ranges from 0 to 24, with higher scores indicating better exercise adherence. In the present study, the EARS-B scale was used to assess exercise adherence among pregnant women, whereas the EARS-C scale was applied to explore its influencing factors. In this study, Cronbach’s alpha of the scale was 0.817.

### 2.6 Pregnancy exercise social support

In the present study, the Physical Activity Social Support Scale (PASSS) was used to assess social support related to physical activity (21). The PASSS consists of 24 items. Each item is rated on a 5-point Likert scale, where one represents “strongly disagree” and five represents “strongly agree.” The total score ranges from 24 to 120, with higher values indicating stronger social support for physical activity. The reported Cronbach’s *α* for this scale was 0.95 (21). In the current study, the Cronbach’s alpha of PASSS was 0.967.

### 2.7 Pregnancy exercise self-efficacy

Prenatal exercise self-efficacy was assessed using the Pregnancy Exercise Self-Efficacy Scale (P-ESES). Kroll et al. originally developed an Exercise Self-Efficacy Scale for individuals with spinal cord injury in 2007 (22). Bland et al. subsequently adapted and validated this scale for pregnant women in 2013, reporting a Cronbach’s *α* of 0.838 (23). The Chinese version of P-ESES was revised by Yang et al., with a Cronbach’s α of 0.804. The P-ESES contains 10 items and is divided into three domains: overcoming exercise barriers, emotional barriers, and support barriers. Each item is rated on a 5-point Likert scale, where one indicates “strongly disagree” and five indicates “strongly agree.” The total P-ESES score ranges from 10 to 50. A total P-ESES score < 20 indicates a low level of exercise self-efficacy, scores from 21 to 40 indicate a moderate level, and scores > 40 indicate a high level of exercise self-efficacy. In the present study, Cronbach’s alpha of this scale was 0.963.

### 2.8 Data analysis

#### 2.8.1 Descriptive analysis

Statistical analyses were performed using SPSS software version 27.0. Necessary tests for normality were conducted using kurtosis and skewness values within the range of −3.29 to 3.29 (24). In this study, descriptive statistics were calculated using the SPSS 27.0 software package. Demographic characteristics and scale scores were summarized using *N* (%), mean (±), and standard deviation (SD) to enhance clarity and interpretability of the results.

#### 2.8.2 Latent profile analysis

Mplus software version 8.3 was employed to conduct a latent profile analysis of pregnancy exercise self-efficacy. The optimal model was determined according to model fit indices and clinical interpretability. Model fit indicators included the Akaike information criterion (AIC), Bayesian information criterion (BIC), adjusted BIC (aBIC), entropy, Lo–Mendell–Rubin likelihood ratio test (LMR), and Bootstrap-based likelihood ratio test (BLRT). Lower values of AIC, BIC, and aBIC indicate better model fit. Entropy values range from 0 to 1 and reflect classification accuracy, with values closer to 1 indicating greater accuracy. The LMR and BLRT tests were applied to compare models with k and (k–1) classes; a value of *p* < 0.05 indicates that the k-class model fits the data significantly better than the (k–1)-class model (25).

#### 2.8.3 Ethical considerations

This study was conducted in accordance with the principles outlined in the Declaration of Helsinki. Ethical approval was obtained from the Ethics Committee of Dongguan Maternal and Child Health Hospital (Approval No. 2024-155), Shunde Women and Children’s Hospital of Guangdong Medical University (KY-2024-070), and Baoan Central Hospital of Shenzhen (BYL20240628). Prior to participation, both oral and written informed consent were obtained from all eligible participants, ensuring that participation or refusal would not influence their work performance or future employment opportunities. In addition, all collected information was anonymized and de-identified to maintain confidentiality. Participants were also informed that they could withdraw from the study at any stage without any consequences.

## 3 Results

### 3.1 Demographic information

In this study, a total of 558 questionnaires were distributed. Among these, 27 questionnaires contained incomplete responses and were therefore considered invalid and excluded from analysis. The remaining questionnaires were complete and regarded as valid, yielding a total of 531 valid responses. Consequently, the effective response rate of the questionnaire was 95.16%. All participants were in the third trimester of pregnancy. Most participants lived in urban areas, and the mean age was 31.81 ± 3.99 years. Only 23.4% of the participants achieved 150 min or more of exercise per week. The mean total PASSS score of the participants was 78.76 ± 18.57, whereas the mean P-ESES score was 34.47 ± 8.10. Additional demographic characteristics are presented in Table 1. Table 2 displays the mean scores for each item of the EARS-C scale (reasons for adherence or non-adherence). Statistical analysis showed that most items exhibited high *F*-values with *p* < 0.05, indicating statistically significant differences across levels of exercise adherence.

**Table 1:** Socio-demographic characteristics of the sample (*N*=531).

**Variable**		**Number**	***N* (%)** **Mean ± SD**
Age		-	31.81±3.99
Pre-pregnancy BMI	Abnormal	169	31.8
	Normal	362	68.2
Gestational weight gain	Abnormal	286	53.9
	Normal	245	46.1
Gravidity	Primigravid	237	44.6
	Multigravid	294	55.4
Parity	Primipara	307	57.8
	Multipara	224	42.2
Planned pregnancy	Intended	402	75.7
	Unplanned	129	24.3
Work nature	Sedentary	281	52.9
	Moderately active	97	18.3
	Home convalescence	153	28.8
Education level	High School and below	89	16.8
	College	138	26.0
	Bachelor's degree or above	304	57.3
Partner's education level	High School and below	103	19.4
	College	132	24.9
	Bachelor's degree or above	296	55.7
Marital Status	Married	515	97.0
	Other	16	3.0
Personal monthly income (RMB)	< 10000	63	11.9
	≥ 10000	468	88.1
Caregiver	Partner	324	61.0
	Other	207	39.0
Residence	Urban	386	72.7
	Other	145	27.3
Exercise habit weekly	< 30min	201	37.9
	< 150min	206	38.8
	≥ 150min	124	23.4
PFMT	Never	355	66.9
	Regularly	176	33.1
PASSS		-	78.76 ± 18.57
P-ESES		-	34.47 ± 8.10

PFMT = Pelvic Floor Muscle Training; PASSS = Physical Activity Social Support Scale; P-ESES = Pregnancy Exercise Self-Efficacy Scale.

**Table 2:** Differences of the EARS-C scale items across pregnancy exercise adherence level (*N* = 531).

Items	Total sample (531)	Profile 1 (233)	Profile 2 (239)	Profile 3 (59)	*F*	*p*
1. I adjust the way I do my exercises to suit myself	1.36 ± 0.85	1.55 ± 0.90	1.21 ± 0.65	1.20 ± 1.19	11.124	<0.001***
2. Other commitments prevent me from doing my exercises	1.76 ± 0.96	1.53 ± 0.85	1.77 ± 0.91	2.63 ± 1.03	34.881	<0.001***
3. I feel confident about doing my exercises	2.45 ± 0.90	1.86 ± 0.84	2.80 ± 0.62	3.36 ± 0.52	153.58	<0.001***
4. I do not have time to do my exercises	2.42 ± 0.99	2.21 ± 1.00	2.39 ± 0.94	3.32 ± 0.60	33.061	<0.001***
5. I’m not sure how to do my exercises	2.22 ± 1.05	2.02 ± 1.05	2.21 ± 0.97	3.08 ± 0.92	26.712	<0.001***
6. I do not do my exercises when I am tired	1.19 ± 0.80	1.01 ± 0.67	1.19 ± 0.71	1.90 ± 1.17	31.896	<0.001***
7. I do my exercises because I enjoy them	2.16 ± 0.96	1.67 ± 0.92	2.48 ± 0.79	2.76 ± 0.84	68.478	<0.001***
8. My family and friends encourage me to do my exercises	2.50 ± 0.95	2.00 ± 1.01	2.79 ± 0.69	3.24 ± 0.60	78.438	<0.001***
9. I stop doing my exercises when my pain is worse	0.82 ± 0.71	0.73 ± 0.69	0.86 ± 0.61	1.00 ± 1.03	4.261	0.015*
10. I do my exercises to improve health	2.66 ± 0.99	2.25 ± 1.10	2.90 ± 0.77	3.32 ± 0.63	46.399	<0.001***

Profile 1, low-adherence group; Profile 2, moderate-adherence group; Profile 3, high-adherence group. *p < 0.05; **p < 0.01; ***p < 0.001. The item scores are presented as mean ± standard deviation (SD).

### 3.2 Results of latent profile analysis

The present study performed latent profile analysis using scores from the EARS-B scale. The model selection was based on several evaluation indicators, including AIC, BIC, aBIC, LMR (*p*-value), and BLRT (*p*-value), to determine the optimal model among 1 to 4 potential profile models constructed sequentially. The model fit indices for each profile are presented in Table 3. As the number of model categories increased from 1 to 4, the AIC, BIC, and aBIC values progressively decreased, while entropy reached the highest value among the four candidate models. However, the LMR result for the 4-profile model was not statistically significant (*p* > 0.05). Therefore, the three-profile model was ultimately selected as the optimal latent profile model for this study, as shown in Table 3.

**Table 3:** Latent profile analysis model fit for exercise adherence in pregnant women (*N* = 531).

Profiles	AIC	BIC	aBIC	LMR (*P*)	BLRT (*P*)	Entropy	Categorical probability
Profile 1	9007.198	9058.495	9020.403	—	—	—	1
Profile 2	8440.484	8521.705	8461.393	<0.001	<0.001	0.816	0.461/0.539
Profile 3	8288.664	8399.808	8317.276	0.0018	<0.001	0.802	0.439/0.450/0.111
Profile 4	7757.399	7898.466	7793.714	0.2101	<0.001	1	0.280/0.403/0.266/0.051

The selected models’ AIC, BIC, and ABIC are information criteria; P-LMR and P-BLRT are model fit statistics. Depiction: Profile 1, low-adherence group; Profile 2, moderate-adherence group; Profile 3, high-adherence group.

### 3.3 Naming of latent profile

**Figure 1:**
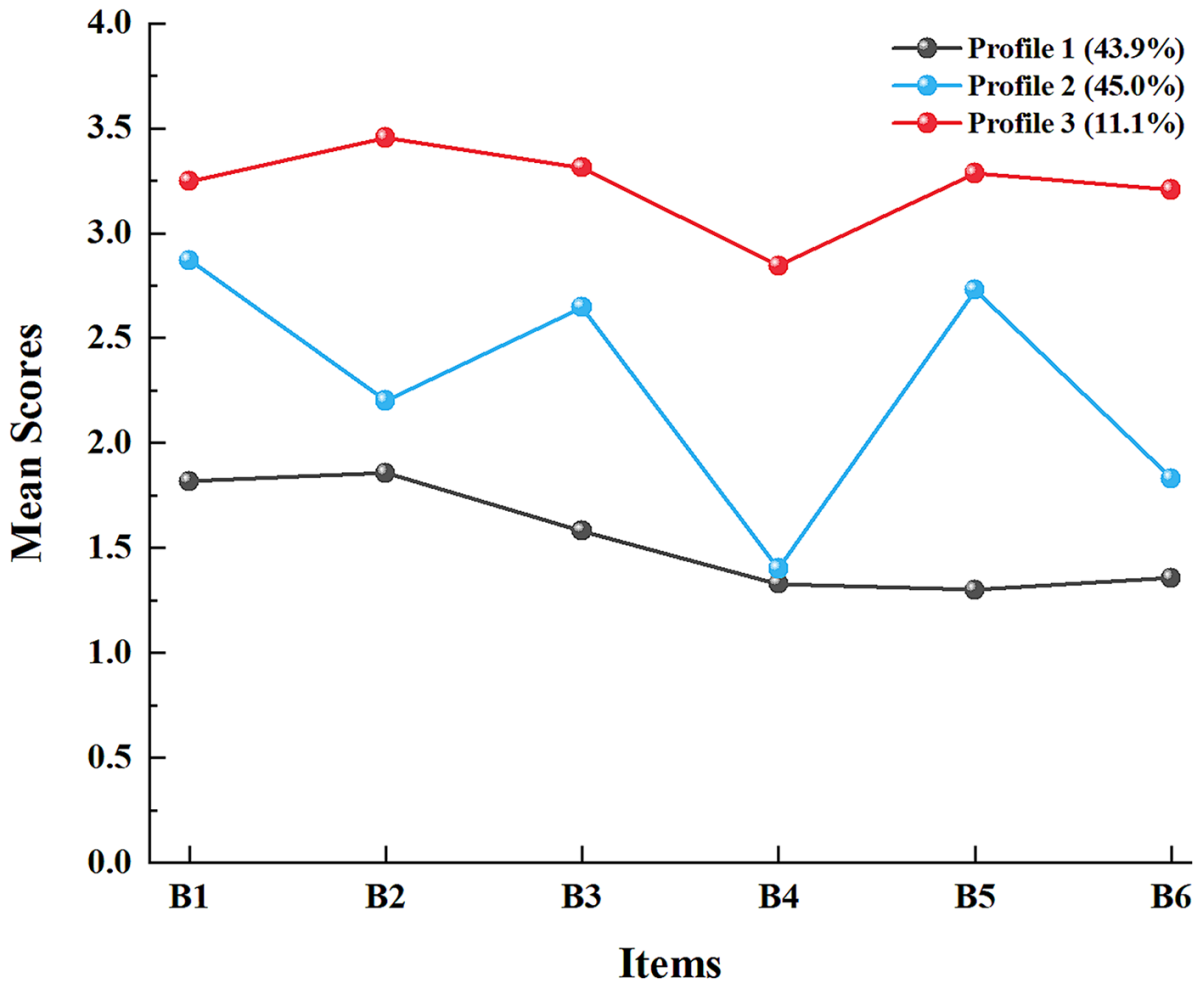
Schematic diagram of the 3-category model of adherence to exercise in pregnant women. Description of the selected LPA profiles of exercise adherence in pregnant women. Depiction: Profile 1 = low-adherence group; Profile 2 = moderate-adherence group; Profile 3 = high-adherence group. Q1 = I do my exercises as often as recommended; Q2 = I do not get around to doing my exercises; Q3 = I do most, or all, of my exercises; Q4 = I do less exercise than recommended by my healthcare professional; Q5 = I fit my exercises into my regular routine; Q6 = I forget to do my exercises in the horizontal coordinate.

Figure 1 illustrates the mean scores of each item across the three exercise adherence profiles among pregnant women. Based on the characteristics of the mean scores across categories, the profiles were labeled as “low-adherence,” “moderate-adherence,” and “high-adherence.” The moderate-adherence profile represented the largest proportion of participants (45.0%), followed by the low-adherence profile (43.9%), whereas the high-adherence profile accounted for 11.1% of participants.

### 3.4 Inter-profile characteristic differences

The chi-square test and one-way analysis of variance were applied to compare differences in potential influencing factors among pregnant women across different exercise adherence profiles. The findings indicated that gravidity, parity, education level, weekly exercise habits, PFMT, exercise self-efficacy, and social support showed statistically significant differences (*p* < 0.05). In contrast, the remaining categorical variables did not demonstrate statistically significant differences (*p* > 0.05), as presented in Table 4.

**Table 4:** Differences in demographics, pregnancy exercise self-efficacy, and pregnancy physical activity support in pregnancy exercise adherence level (*N* = 531).

Variable	Profile 1 (233)	Profile 2 (239)	Profile 3 (59)	*χ2 / F*	*p*
Age	31.47 ± 4.12	32.09 ± 3.85	32.02 ± 3.95	1.538	0.216
EARS-B	9.32 ± 2.59	13.77 ± 1.96	19.66 ± 2.15	553.6111	<0.001***
PASSS	67.12 ± 16.25	86.14 ± 13.60	94.83 ± 17.45	128.479	<0.001***
P-ESES	29.10 ± 7.56	37.90 ± 5.52	41.80 ± 5.18	151.931	<0.001***
Pre-pregnancy BMI					
Abnormal	81 (34.80%)	73 (30.50%)	15 (25.40%)	2.223	0.329
Normal	152 (65.20%)	166 (69.50%)	44 (74.60%)		
Gestational weight gain					
Abnormal	133 (57.10%)	127 (53.10%)	26 (44.10%)	3.300	0.192
Normal	100 (42.90%)	112 (46.90%)	33 (55.90%)		
Gravidity					
Primigravid	120 (51.50%)	89 (37.20%)	28 (47.50%)	9.928	0.007**
Multigravid	113 (48.50%)	150 (62.80%)	31 (52.50%)		
Parity					
Primipara	151 (64.80%)	118 (49.40%)	38 (64.40%)	12.706	0.002**
Multipara	82 (35.20%)	121 (50.60%)	21 (35.60%)		
Planned pregnancy					
Intended	168 (72.10%)	185 (77.40%)	49 (83.10%)	3.751	0.153
Unplanned	65 (27.90%)	54 (22.60%)	10 (16.90%)		
Work nature					
Sedentary	123 (52.80%)	125 (52.30%)	33 (55.90%)	0.998	0.910
Moderately active	43 (18.50%)	42 (17.60%)	12 (20.30%)		
Home convalescence	67 (28.80%)	72 (30.10%)	14 (23.70%)		
Education level					
High School and below	40 (17.20%)	42 (17.60%)	7 (11.90%)	11.353	0.023*
College	60 (25.80%)	71 (29.70%)	7 (11.90%)		
Bachelor’s degree or above	133 (57.10%)	126 (52.70%)	45 (76.30%)		
Partner’s education level					
High School and below	50 (21.50%)	46 (19.20%)	7 (11.90%)	2.793	0.593
College	57 (24.50%)	59 (24.70%)	16 (27.10%)		
Bachelor’s degree or above	126 (54.10%)	134 (56.10%)	36 (61.00%)		
Marital status					
Married	223 (95.70%)	235 (98.30%)	57 (96.60%)	2.800	0.247
Other	10 (4.30%)	4 (1.70%)	2 (3.40%)		
Personal monthly income (RMB)					
<10,000	31 (13.30%)	27 (11.30%)	5 (8.50%)	1.184	0.553
≥10,000	202 (86.70%)	212 (88.70%)	54 (91.50%)		
Caregiver					
Partner	153 (65.70%)	133 (55.60%)	38 (64.40%)	5.297	0.071
Other	80 (34.30%)	106 (44.40%)	21 (35.60%)		
Residence					
Urban	159 (68.20%)	179 (74.90%)	48 (81.40%)	5.142	0.076
Other	74 (31.80%)	60 (25.10%)	11 (18.60%)		
Exercise habit weekly					
<30 min	117 (50.20%)	78 (32.60%)	6 (10.20%)		
<150 min	81 (34.80%)	107 (44.80%)	18 (30.50%)	62.962	0.001***
≥150 min	35 (15.00%)	54 (22.60%)	35 (59.30%)		
PFMT					
Never	177 (76.00%)	152 (63.60%)	26 (44.10%)	23.697	<0.001***
Regularly	56 (24.00%)	87 (36.40%)	33 (55.90%)		

PFMT, pelvic floor muscle training; PASSS, physical activity social support scale; P-ESES, pregnancy exercise self-efficacy scale; Depiction: Profile 1 = low-adherence group; Profile 2 = moderate-adherence group; Profile 3 = high-adherence group. *p < 0.05; **p < 0.01; ***p < 0.001.

### 3.5 Three profile-related differences in P-ESES and P-PASSS scores

**Figure 2:**
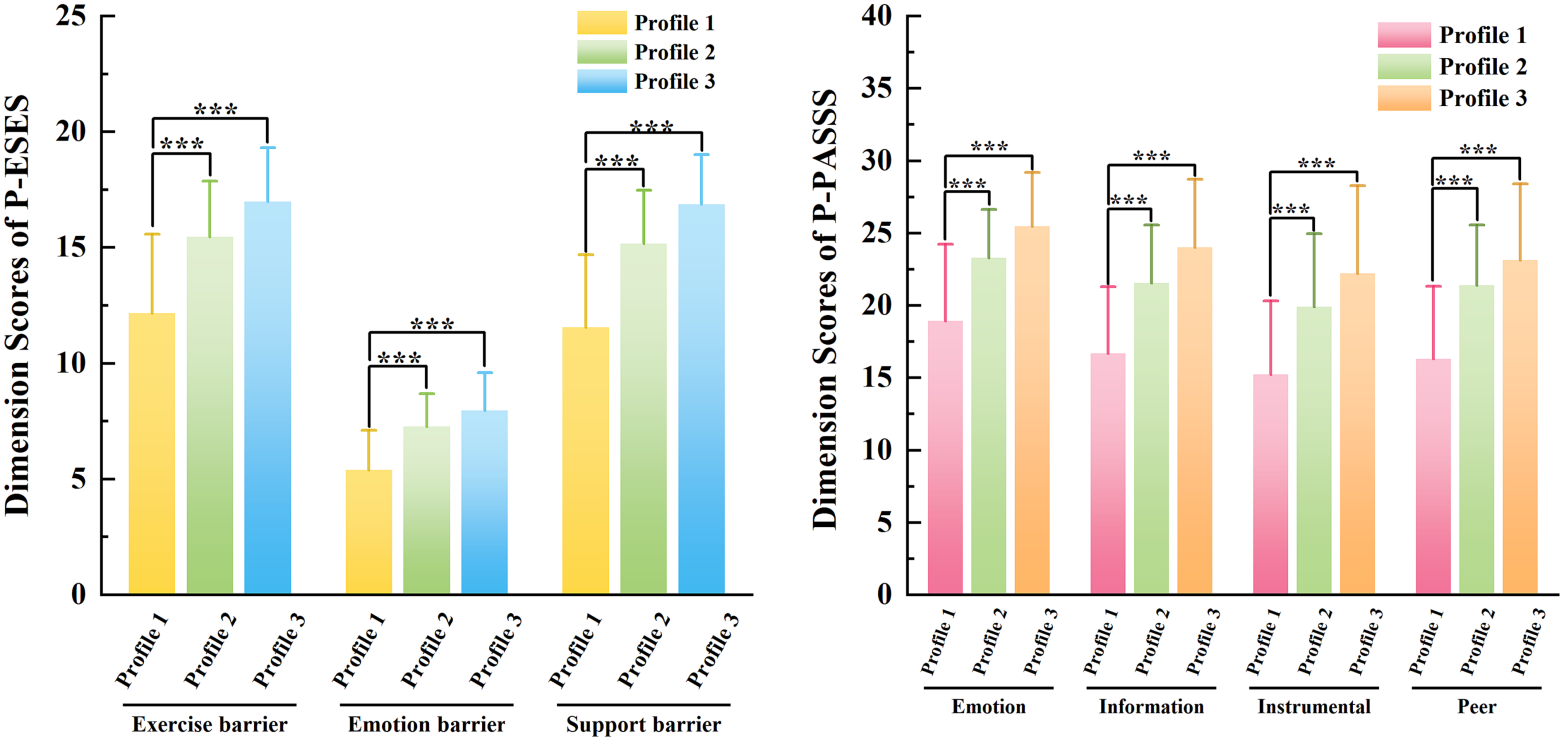
P-ESES and P-PASSS dimension scores across three profiles. Reference group: Profile 1. Values presented are mean ± SD. *p < 0.05, **p < 0.01, ***p < 0.001 (by Tukey’s HSD test). Depiction: P-ESES, Pregnancy Exercise Self-Efficacy Scale; P-PASSS, Pregnancy Physical Activity Social Support Scale; Profile 1 = low-adherence group; Profile 2 = moderate-adherence group; Profile 3 = high-adherence group.

Figure 2 illustrates the scores of each dimension of P-ESES and P-PASSS across the three profiles and highlights the differences between groups. The left panel shows that Profile 1 exhibited the lowest P-ESES scores across all dimensions, whereas Profile 3 demonstrated the highest values across these dimensions. Compared with Profile 1, statistically significant differences (*p* < 0.05) were observed in all other profiles across each dimension.

The right panel indicates that Profile 1 had the lowest P-PASSS scores among the three profiles, while Profile 3 showed the highest scores across all dimensions. Using Profile 1 as the reference group, all other profiles demonstrated statistically significant differences (*p* < 0.05) in every dimension.

### 3.6 Multinomial logistic regression of exercise adherence profiles

#### 3.6.1 Demographic information

Table 5, when the low-adherence profile was used as the reference category, relative to the moderate-adherence profile, weekly exercise habit < 30 min (OR = 0.218, *p* < 0.001) was significantly negatively associated with the moderate-adherence profile. In contrast, PASSS scores (OR = 1.060, *p* < 0.001) and P-ESES scores (OR = 1.189, *p* < 0.001) showed significant positive associations with the moderate-adherence profile. These results suggest that women who exercise less than 30 min per week are less likely to be classified into the moderate-adherence profile. Additionally, higher PASSS and P-ESES scores increase the likelihood of belonging to the moderate-adherence group.

**Table 5:** Multinomial logistic regression analysis of the pregnancy exercise adherence among three profiles (*N* = 531).

**Variables**	**Profile 1**^**a**^ **vs. Profile 2**				**Profile 1**^**a**^ **vs. Profile 3**				**Profile 2**^**a**^ **vs. Profile 3**			
	**β**	**SE**	***P***	**OR**	**β**	**SE**	***P***	**OR**	**β**	**SE**	***P***	**OR**
	-9.155	0.995	<0.001***		-16.820	1.800	<0.001***		-7.666	1.555	<0.001***	
Gravidity												
Primigravid	-0.652	0.405	0.107	0.521	-0.862	0.616	0.162	0.422	-0.209	0.507	0.680	0.811
Parity												
Primipara	0.057	0.406	0.889	1.059	0.805	0.632	0.202	2.237	0.748	0.521	0.151	2.113
Education level												
High School and below	0.729	0.380	0.055	2.073	0.678	0.615	0.271	1.969	-0.051	0.518	0.921	0.950
College	0.285	0.301	0.343	1.330	-0.924	0.553	0.095	0.397	-1.208	0.484	0.013**	0.299
Exercise habits weekly												
<30min	-1.525	0.384	<0.001***	0.218	-3.855	0.636	<0.001***	0.021	-2.330	0.539	<0.001***	0.097
<150min	-0.397	0.366	0.279	0.673	-1.747	0.493	<0.001***	0.174	-1.351	0.375	<0.001***	0.259
PFMT												
Never	-0.554	0.286	0.053	0.574	-1.142	0.434	0.009**	0.319	-0.587	0.358	0.100	0.556
PASSS	0.059	0.010	<0.001***	1.060	0.087	0.015	<0.001***	1.091	0.028	0.013	0.025**	1.029
P-ESES	0.174	0.024	<0.001***	1.189	0.298	0.042	<0.001***	1.347	0.124	0.037	<0.001***	1.133

^a^Reference group; Profile 1, low-adherence group; Profile 2, moderate-adherence group; Profile 3, high-adherence group; PFMT, pelvic floor muscle training; PASSS, physical activity social support scale; P-ESES, pregnancy exercise self-efficacy scale; *p < 0.05; **p < 0.01; ***p < 0.001.

Relative to the high-adherence profile, weekly exercise habit < 30 min (OR = 0.021, *p* < 0.001), weekly exercise < 150 min (OR = 0.174, *p* < 0.001), and never performing PFMT (OR = 0.319, *p* < 0.05) were significantly negatively associated with the high-adherence profile. Conversely, PASSS scores (OR = 1.091, *p* < 0.001) and P-ESES scores (OR = 1.347, *p* < 0.001) were significantly positively associated with high adherence. These findings indicate that, first, women who exercise < 150 min and those who have never engaged in PFMT are less likely to be categorized as high-adherence. Furthermore, higher PASSS and P-ESES scores increase the likelihood of being categorized into the high-adherence group.

When the moderate-adherence profile was used as the reference category, relative to the high-adherence profile, college education level (OR = 0.299, *p* < 0.05), weekly exercise habit < 30 min (OR = 0.097, *p* < 0.001), and weekly exercise < 150 min (OR = 0.259, *p* < 0.001) were significantly negatively associated with the high-adherence profile. In contrast, PASSS scores (OR = 1.029, *p* < 0.05) and P-ESES scores (OR = 1.133, *p* < 0.001) showed significant positive associations with the high-adherence profile. These results indicate that pregnant women with college-level education and lower weekly exercise durations (< 30 min or < 150 min) are less likely to be categorized in the high-adherence profile. Meanwhile, higher PASSS and P-ESES scores increase the probability of classification into the high-adherence group.

#### 3.6.2 Reasons for adherence or non-adherence

Table 6, when the low-adherence profile was used as the reference group, relative to the moderate-adherence profile, item 1 (“I adjust the way I do my exercises to suit myself”; OR = 0.689, *p* < 0.05) showed a significant negative association with moderate adherence. In contrast, item 2 (“Other commitments prevent me from doing my exercises”; OR = 1.520, *p* < 0.05), item 3 (“I feel confident about doing my exercises”; OR = 3.280, *p* < 0.001), item 7 (“I do my exercises because I enjoy them”; OR = 1.981, *p* < 0.001), item 8 (“My family and friends encourage me to do my exercises”; OR = 1.513, *p* < 0.05), and item 10 (“I do my exercises to improve my health”; OR = 1.412, *p* < 0.05) demonstrated significant positive associations with moderate adherence. These findings indicate that higher scores for items 3, 7, 8, and 10 increase the likelihood of belonging to the moderate-adherence profile, whereas lower scores for item 1 are more likely to be associated with the moderate-adherence category.

**Table 6:** Multinomial logistic regression analysis of the EARS-C scale items among three profiles (*N* = 531).

Variables	Profile 1^a^ vs. Class 2				Profile 1^a^ vs. Class 3				Profile 2^a^ vs. Class 3			
	β	SE	*P*	OR	β	SE	*P*	OR	β	SE	*P*	OR
	−7.180	0.854	<0.001***		−21.887	2.417	<0.001***		−14.707	2.271	<0.001***	
1. I adjust the way I do my exercises to suit myself	−0.372	0.170	0.028**	0.689	−0.496	0.288	0.085	0.609	−0.124	0.246	0.616	0.884
2. Other commitments prevent me from doing my exercises	0.418	0.167	0.012**	1.520	0.695	0.262	0.008**	2.005	0.277	0.218	0.205	1.319
3. I feel confident about doing my exercises	1.166	0.193	<0.001***	3.208	2.091	0.445	<0.001***	9.089	0.925	0.413	0.025**	2.522
4. I do not have time to do my exercises	0.140	0.153	0.359	1.150	1.505	0.439	<0.001***	4.505	1.365	0.419	0.001***	3.916
5. I’m not sure how to do my exercises	0.112	0.140	0.424	1.119	0.445	0.283	0.116	1.561	0.333	0.258	0.196	1.395
6. I do not do my exercises when I am tired	0.197	0.192	0.307	1.217	0.990	0.289	<0.001***	2.692	0.794	0.234	<0.001***	2.211
7. I do my exercises because I enjoy them	0.684	0.145	<0.001***	1.981	0.761	0.267	0.004**	2.141	0.077	0.238	0.745	1.080
8. My family and friends encourage me to do my exercises	0.414	0.165	0.012**	1.513	1.320	0.423	0.002**	3.743	0.906	0.401	0.024**	2.474
9. I stop exercising when my pain is worse	0.161	0.196	0.414	1.174	0.631	0.379	0.096	1.880	0.471	0.342	0.168	1.602
10. I do my exercises to improve my health	0.345	0.157	0.027**	1.412	0.564	0.325	0.083	1.757	0.218	0.300	0.466	1.244

a Reference group; Profile 1 = low-adherence group; Profile 2 = moderate-adherence group; Profile 3 = high-adherence group; *p < 0.05; **p < 0.01; ***p < 0.001.

Relative to the high-adherence profile, item 2 (“Other commitments prevent me from doing my exercises”; OR = 2.005, *p* < 0.05), item 3 (“I feel confident about doing my exercises”; OR = 9.089, *p* < 0.001), item 4 (“I do not have time to do my exercises”; OR = 4.505, *p* < 0.001), item 6 (“I do not do my exercises when I am tired”; OR = 2.692, *p* < 0.001), item 7 (“I do my exercises because I enjoy them”; OR = 2.141, *p* < 0.05), and item 8 (“My family and friends encourage me to do my exercises”; OR = 3.743, *p* < 0.05) showed significant positive associations. These results suggest that higher scores for items 2, 3, 4, 6, 7, and 8 increase the likelihood of being categorized into the high-adherence profile.

When the moderate-adherence profile was used as the reference group, relative to the high-adherence profile, item 3 (“I feel confident about doing my exercises”; OR = 2.522, *p* < 0.05), item 4 (“I do not have time to do my exercises”; OR = 3.916, *p* < 0.001), item 6 (“I do not do my exercises when I am tired”; OR = 2.211, *p* < 0.001), and item 8 (“My family and friends encourage me to do my exercises”; OR = 2.474, *p* < 0.05) were significantly positive. This suggests that the higher scores of items 3, 4, 6, and 8 are more likely to be categorized as high adherence.

## 4 Discussion

The present study is the first to investigate latent profiles of exercise adherence among women in the third trimester using a person-centered analytical approach. Exercise adherence among pregnant women was classified into three distinct profiles: low-adherence (43.9%), moderate-adherence (46.0%), and high-adherence (11.1%). The mean adherence scores for these three groups were 9.32, 13.77, and 19.66, respectively. These findings were consistent with those reported by Lu et al. (26). The mean score on the EARS-B scale among pregnant women was 12.47 ± 4.01, which remained below the established cut-off value (27).

The findings of the present study indicated that education level, weekly exercise habits, and PFMT were associated with exercise adherence among pregnant women. Consistent with previous research, lower educational attainment was identified as a negative factor in achieving recommended levels of physical activity during pregnancy (28, 29). Compared with the high-adherence group, pregnant women with lower educational attainment were more likely to be classified into the moderate-adherence group. Zhang et al. (30) reported that pre-pregnancy exercise habits were a significant predictor of the intention to initiate physical activity. In the present study, women who performed PFMT were more likely to demonstrate higher exercise adherence. Pelvic floor disorders, such as stress urinary incontinence (SUI) and pelvic floor pain during late pregnancy, may negatively affect women’s motivation and ability to participate in exercise. PFMT has been shown to be one of the most effective interventions for managing SUI through strengthening pelvic floor muscles. Physical activity programs that integrate PFMT may therefore provide additional motivation for pregnant women to participate in aerobic exercise during pregnancy (31, 32). Moreover, information regarding the benefits of exercise and the risks associated with physical inactivity during pregnancy should be incorporated into tailored exercise programs, particularly for pregnant women with lower levels of formal education.

Multinomial logistic regression analysis indicated that exercise self-efficacy was an important factor influencing exercise adherence among pregnant women; stronger self-efficacy significantly increased the likelihood of belonging to the moderate- or high-adherence profiles (Tables 5, 6). Moreover, women in Profile 3 showed significantly higher P-ESES scores across all dimensions than those in Profiles 1 and 2 (Table 4; Figure 2). Exercise self-efficacy refers to pregnant women’s confidence in their capability to perform exercise (33). According to Bandura’s self-efficacy theory, exercise self-efficacy is a key determinant for initiating and sustaining new health behaviors (34, 35). A systematic review also demonstrated that regular physical activity was positively associated with higher exercise self-efficacy. Pregnant women who possessed stronger exercise self-efficacy tended to show more positive attitudes toward physical activity and exercise during pregnancy and were therefore more likely to adhere to exercise recommendations (36). In the present study, only 11.86% of participants demonstrated high exercise self-efficacy. These findings are consistent with those reported by Chen et al. (37), who observed that only 12.70% of participants in China exhibited good exercise self-efficacy. This consistently low level is likely attributable to the unique challenges of late pregnancy. With increasing gestational age, women undergo multiple physical changes, including weight gain (38), lumbopelvic pain (39), and poor sleep quality (40). In addition, close to the time of delivery, many pregnant women experience negative emotions such as fear of childbirth (41). These physical and psychological changes make it difficult for pregnant women to believe that they can achieve exercise goals (56). In addition, the experience of failing to reach exercise goals may further undermine pregnant women’s self-efficacy, thereby reducing their exercise adherence (34). Therefore, interventions targeting pregnant women with low exercise self-efficacy should integrate multifaceted strategies to build confidence and capability. These interventions may include: first, strengthening guidance on prenatal exercise; second, helping pregnant women develop detailed exercise plans; third, prompting pregnant women to adjust their exercise routines to match their abilities (42, 43).

Similarly, women with higher P-PASSS scores in this study were more likely to be categorized into Profiles 2 or 3. Women in Profile 3 demonstrated significantly higher P-PASSS scores across all dimensions compared with those in Profiles 1 and 2 (Table 4; Figure 2). These findings are consistent with a large body of research indicating that social support is an important predictor of physical activity during pregnancy (44–46). Self-efficacy theory could explain these results. As an important external factor, social support enhanced pregnant women’s exercise confidence through vicarious experience (e.g., observing peers’ successful exercise) and verbal persuasion (e.g., encouragement from family) (34). In turn, this enhanced confidence may indirectly improve exercise adherence. Studies by Lu and Xiao (37, 47) indicated that physical activity self-efficacy mediates the association between social support and physical activity. Indeed, partners, family members, friends, and healthcare professionals have consistently been identified as major sources of social support that encourage physical activity during pregnancy (48). Makaruk et al. (49) found that, compared to other populations, pregnant women are more empathic to each other, and group exercise with other pregnant women makes them feel more belonging. Therefore, pregnant women should be encouraged to participate in group training. Shared experiences and mutual encouragement within the group play a key role in improving exercise adherence. In addition, partners should be encouraged to exercise together with pregnant women (50).

The EARS-C scale was used to explore the reasons for adherence among women with both good and poor exercise adherence. The results identified items 3 and 8 as two key protective factors in the moderate- and high-adherence groups (Table 6), indicating that exercise self-efficacy and social support play crucial roles in maintaining exercise adherence (23). Furthermore, enjoyment of exercise is also an additional facilitating factor. Consistent with social cognitive theory, self-efficacy as a personal factor and social support as a social environmental factor may jointly influence pregnant women’s exercise adherence behavior (51). In addition, enjoyment of exercise can be viewed as a positive affective experience that may enhance self-efficacy or positive outcome expectations, thereby indirectly promoting exercise adherence. Accordingly, prenatal exercise programs should emphasize enjoyment, skill mastery, and a sense of achievement while simultaneously promoting maternal well-being and vitality. Mobile health (mHealth) exercise programs that incorporate attractive course visuals and motivating music may enhance the overall exercise experience and increase enjoyment (52). Furthermore, healthcare professionals should focus on developing personalized and gamified mHealth exercise programs specifically designed for pregnant women.

Several barriers to exercise adherence were also identified, including competing commitments, lack of time, and fatigue, which are consistent with findings reported by Shang et al. (11) and Koleilat et al. (53). Previous research has suggested that these barriers are largely related to the dual responsibilities of caregiving and employment faced by many women (48). Following the implementation of China’s “three-child policy,” the proportion of multiparous women has increased. In the present study, more than half of the multiparous participants experienced difficulties balancing competing responsibilities with physical exercise. Therefore, time constraints should be carefully considered when designing physical activity interventions for pregnant women. In response, a community-based program is proposed that would facilitate peer interaction and provide opportunities for sharing common challenges and experiences (54). Such programs could include weekly group exercise sessions adapted to different fitness levels, together with workshops addressing time management and strategies for balancing multiple responsibilities (55).

This study has two main strengths. First, a multicenter design was adopted, which reduces the bias often observed in single-center studies. Second, a person-centered analytical approach was used to capture heterogeneous adherence patterns among individuals. However, several limitations should be noted. First, most variables were collected using self-reported questionnaires, with the exception of weight and height. Nevertheless, the scales used in this study demonstrated good reliability and validity, supporting the accuracy of the findings. In addition, the cross-sectional design limits causal inference and does not allow examination of how exercise adherence changes throughout the entire pregnancy period. Due to limitations of research resources and time, this study employed convenience sampling, which restricts the generalizability of the sample. Future research should therefore follow pregnant women across all three trimesters using longitudinal designs to determine causality and adopt random sampling to improve generalizability.

## 5 Conclusion

The present study demonstrated that exercise adherence among pregnant women was heterogeneous and could be categorized into three distinct profiles. Exercise self-efficacy, social support, weekly exercise habits, PFMT, enjoyment of exercise, competing commitments, lack of time, and fatigue were identified as major factors influencing sustained exercise. These findings provide an important foundation for the development of future tailored physical activity interventions for pregnant women.

## Data Availability

The datasets presented in this article are not readily available because data sharing is restricted by ethical approval to protect participant confidentiality, so the dataset is not available to others. Requests to access the datasets should be directed to Chenhui Zhou, chenhuizh2026@126.com.
